# Implementation and effectiveness of advance care planning in hospitalized older adults with chronic heart failure: a mixed-methods systematic review and meta-analysis

**DOI:** 10.3389/fmed.2025.1566977

**Published:** 2025-04-29

**Authors:** Li Chen, Yuqiu Cheng, Jun Qu, Zhangyi Wang

**Affiliations:** ^1^School of Nursing, Hunan Normal University & Affiliated Hengyang Central Hospital, Changsha, Hunan, China; ^2^School of Nursing, Kiang Wu Nursing College of Macao, Cotai, Macao SAR, China

**Keywords:** advance care planning, heart failure, meta-analysis, systematic review, older adults

## Abstract

**Objectives:**

This study aims to integrate the data on the effects of a pre-established medical care program on hospitalized older adults with chronic heart failure (CHF).

**Method:**

A comprehensive systematic review incorporating mixed research methodologies was undertaken. Quality assessment was conducted using the Critical Appraisal Tool developed by Joanna Briggs Institute, adhering to the PRISMA guidelines for studies. Where appropriate, data were synthesized and aggregated for meta-analysis or meta-aggregation.

**Results:**

A total of 2,825 articles were found, of which 11 met the inclusion criteria. Meta-analysis showed that the implementation of advance care planning (ACP) can significantly increase the willingness and proportion of patients with CHF to choose and receive hospice services during their end-of-life phase. Meta-aggregation showed that the ACP intervention has a positive impact on participants, promotes their knowledge and understanding, and makes them share their decision-making with their families.

**Conclusion:**

ACP is a promising and feasible intervention that can help older adults with CHF accurately understand ACP and express their wishes timely. This study provides insights and empirical evidence to improve ACP, and valuable guidance and reference for future clinical practice.

**Systematic review registration:**

https://www.crd.york.ac.uk/PROSPERO/, PROSPERO, identifier: CRD42024580814.

## 1 Introduction

As the population ages and the incidence of chronic diseases increases, medical and social healthcare systems are facing an increasingly heavy burden (1). Chronic diseases are illnesses that persist for a long time and are rarely cured, which generally need long-term treatment and monitoring, and hospitalization is always possible (2, 3). Previous studies have shown that heart failure (HF) is a significant contributor to hospital admissions and that HF patients usually have a high comorbidity rate, which can lead to a significant disease burden (4, 5). Estimates suggest that total healthcare costs due to HF in the USA will reach $69.8 billion by 2030, which equates to a cost of approximately $244 per US adult (6). Although the lifespan of HF patients has increased of late, their quality of life has not significantly improved (7, 8). Consequently, HF imposes economic and life burden on the family, which makes it a significant worldwide health burden (9, 10).

Chronic heart failure (CHF) is rapidly becoming a major threat to human health (11) and is one of the primary causes of hospitalization in the older adults (10). Clinical manifestations of CHF usually include dyspnea, edema, fluid retention, and fatigue. Patients with CHF are more likely to develop other comorbidities (12). Cardiogenic shock (CS) is an extremely serious clinical condition, and studies have shown that more than half of CS cases may be closely associated with CHF (13). In addition, patients with CHF often suffer from complications due to psychological disorders, which not only further severely impair their quality of life but also significantly increase the mortality rate, perhaps even doubling it (14). Furthermore, CHF patients have high rates of rehospitalization within 2 years and in-hospital mortality, so approaches to alleviating unnecessary burdens need to be developed (15).

Advance care planning (ACP) is a comprehensive and ongoing process in which the individual is actively involved in making decisions about their future healthcare, with a special emphasis on ensuring that they receive care in accordance with their preferences, values, and philosophy of life, especially in the face of a serious illness or at the end of life (EOL). This approach enables the individual to express their wishes and preferences regarding their healthcare, thereby safeguarding their autonomy even when they may lose the capacity to do so directly (16). Previous studies have shown that ACP interventions can improve outcomes in older patients, including making specific decisions together with relatives, identifying wishes and values, and reducing unnecessary treatment and hospitalization (17–19). However, promoting ACP remains challenging, especially among CHF patients who have conscious disturbances due to the changes in their illness (e.g., CS), which makes it impossible for the patients to accurately convey their wishes (20). Therefore, timely ACP interventions are necessary for patients with CHF (21).

Several systematic assessments have been conducted to evaluate the efficacy of ACP programs in different groups of older patients (22–25). However, none of these assessments have focused on ACP for older adults with CHF. Therefore, this mixed-methods systematic review (MMSR) aims to bring together relevant evidence from both quantitative and qualitative studies using a holistic approach, taking into account the importance of validity and practical experience (26). In an MMSR, the quantitative component incorporates a wide range of research design options, whereas the qualitative component explores the meaning and understanding of interventions. In this study, a definitive synthesis study combining quantitative and qualitative evidence was designed to provide strategic guidance for the design and implementation of ACP for older populations with CHF.

## 2 Method

This review followed the MMSR methodological framework developed by Joanna Briggs Institute (JBI) and Preferred Reporting Items for Systematic Reviews and Meta-Analyses (PRISMA) and aimed to accurately address the established review questions and systematically synthesize and integrate the data (27, 28). This review was registered on PROSPERO (CRD42022329615).

### 2.1 Search strategy

Seven databases were used in the search, namely PubMed, CINAHL, MEDLINE, Web of Science, CNKI, Embase, and Cochrane Library, which was carried out from the date of initiation of the database to 29 October 2024. The search keywords encompassed relevant subject headings and universal symbols related to “advance care planning,” “end of life discuss,” “heart failure,” and “aged.” The gray literature database and clinical trial registries were searched for relevant unpublished studies. Reference lists of the included studies and relevant review articles were manually searched to identify eligible studies that may have been overlooked during the initial search. A detailed search plan was developed and documented prior to the beginning of the search to ensure that the search was comprehensive and reproducible. To mitigate the risk of publication bias, searches were not restricted by publication status.

### 2.2 Study selection

The inclusion criteria were developed based on participants, interventions, outcomes/associated phenomena, context, and type of study. Interventions that focused on the development of resuscitation-assisted euthanasia were excluded, but interventions that focused on ACP or advance directive (AD) forms were included. ACP is an ongoing process and should therefore report its relevant process outcomes (including various outcomes in the progress of the ACP as well as the final outcome) (22, 29). Coronary care unit (CCU) patients were excluded because they could not make clear decisions (30). Average age was set as 50 years to cater to a sufficient number of included studies and to improve the reliability of the results (Table 1). Two reviewers independently screened the title and abstract of the studies to exclude those that were not relevant to the present study, and potentially compatible studies were retained for further evaluation. One reviewer first meticulously browsed and reviewed the full text of an initially included study according to the eligibility criteria, followed by an independent review by another reviewer. In case of any disagreement, an in-depth discussion was held to reach consensus and make a decision.

**Table 1:** Eligibility criteria of studies.

**Criteria Dimensions**	**Inclusion**		**Exclusion**
	**Quantitative component**	**Qualitative component**	**Quantitative and qualitative components**
Types of participants	Patients diagnosed with HF. Older adults (≥50 years old), regardless of gender and geographical location		Mean age <50 years, the patient was diagnosed with other chronic diseases
Types of interventions	Adopt alternative measures using any tools or methods to promote the spread of ACP or AD With or without a comparison group		Interventions to help develop resuscitation-assisted euthanasia
Outcomes/phenomena of interest	ACP outcomes ACP process outcomes, such as knowledge, readiness, preference, and self-efficacy ACP outcomes, such as ACP engagement and completion of ACP	Experiences with the interventions	
Context	Community, hospital settings, clinics, or homes		CCU
Types of studies	1. Various experimental studies: RCTs, non-RCTs, observational studies (prospective studies, retrospective cohort studies, cross-sectional studies) 2. In mixed-methods studies, quantitative data can be extracted	1. Various qualitative studies 2. In mixed-methods studies, qualitative data can be extracted	Review, guidelines, case reports, study proposals/protocols, conference abstracts, PhD theses, and non-peer-reviewed journals

ACP, advance care planning; AD, advance directive; CCU, cardiac care unit; RCT, randomized controlled trial.

### 2.3 Data extraction

Using standardized JBI data extraction methods, one reviewer extracted quantitative and qualitative data separately and discussed the methods and findings with the other reviewer (31). The quantitative data extracted included information on authors, published year, participants, environmental context, study design, interventions, findings, and effectiveness (Table 2). The qualitative data extracted included information on the phenomenon of interest related to the research objectives, study population, research methodology, contextual environment, cultural information, and data analyses (Table 3). In addition, an interpretation of the results of qualitative data analysis was obtained from the authors, which encompassed the primary theme of cultural literacy and its subthemes. Two reviewers independently assessed the “confidence” (defined as clarity, reliability, and unsupported) of the study information based on the established descriptions (specifically, direct quotations from participants' original statements, field observation records, or other raw data) (31).

**Table 2:** Summary of characteristics of quantitative results of the included studies.

**References**	**Participants and setting**	**Design**	**Intervention**	**Results**	**Outcome measures**
Schellinger et al. (32)	CHF patients (*n =* 1,894) Medical center	RCT	DS-ACP program	A chart audit revealed 94.3% of those completing the DS-ACP process had a health directive compared to 24.8% of non-completers (*p < * 0.001). Of the patients who died by the end of the study period (*n =* 286), DS-ACP participants were more likely to have used hospice compared with non-participants (56% vs. 37%, *p =* 0.002)	The completion of AD forms Preferences for EOL care
Evangelista et al. (33)	CHF patients (*n =* 36) Medical center	Prospective study	Palliative care consultation, 3 months	Average scores on the ADAS increased from 57% to 80% (*p < * 0.001) from baseline to 3 months. The number of participants who completed ADs increased (28% vs. 47%, *p =* 0.016)	ADAS The completion of AD forms
Sadeghi et al. (34)	CHF patients (*n =* 37) Medical center	Prospective study	ACP educational video about shared decision-making (< 15 min), 6 months	The number of patients having a signed scanned POLST form increased from 10 (27%) before the intervention to 16 (43%) 6 months after the intervention (*p =* 0.03). 49% of patients had signed AD or POLST forms in their medical records 6 months after the index hospitalization compared with 36% before the intervention *(p =* 0.06)	The completion of AD and POLST forms
El-Jawahri et al. (35)	CHF patients (*n =* 246) Medical center	RCT	ACP video decision support tool (6 min), 3 months	More participants in the video-assisted intervention arm preferred to forgo CPR and intubation (68% and 76%, respectively) compared with those in the verbal control arm (35% and 48%, *p < * 0.001 and *p < * 0.001, respectively). Participants in the video-assisted intervention arm had higher mean ACP knowledge scores compared with the control participants (4.1 ± 1.4 vs. 3.0 ± 1.5; *p < * 0.001)	Preferences for EOL care Knowledge of ACP
Metzger et al. (36)	CHF patients (*n =* 29) Medical center	RCT	SPIRIT-HF (44-96 min)	The SPIRIT-HF group showed higher improvement, with 5 dyads congruent at baseline in each group, and 13 out of 14 congruent at time 2 in the SPIRIT-HF group; and 9 out of 15 congruent in the control group. The difference between the groups was not statistically significant (*p =* 0.064). There were no significant differences between the groups in patient decisional conflict or surrogate decision-making confidence. Intervention dyads were nearly nine times as likely as controls to achieve congruence in patients' GOC	GOC DCS DMC
Malhotra et al. (37)	CHF patients (*n =* 200) Medical center	RCT	ACP intervention, 2 years	Patient preference for aggressive EOL care was lower for older patients (OR = 0.96, *p < * 0.001). Overall, 64% (*n* = 128) of patients changed their stated preference for type of EOL care at least once through the study period	Change in preferences for EOL care
Ahluwalia et al. (38)	CHF patients (*n =* 20) Medical center	Mixed methods	ACP group visit (90 min), 1 month	Patient readiness to engage in ACP improved significantly from pre- to post-group visit (change score +0.53; *p < * 0.01) but dropped almost back to pre-group visit levels by the 1-month follow-up survey (change score−0.52; *p < * 0.01). Patient self-efficacy did not significantly change overall from pre- to post-group visit (change score +0.29; *p =* 0.11) and declined to below pre-group visit levels by the 1-month assessment (change score 0.40; *p < * 0.05)	ACP engagement ACP-relevant outcomes
Coster et al. (39)	CHF patients (*n =* 30) Medical center	Prospective study	ACP conversation	Most patients (78%) did not want to be readmitted and preferred to die at home. Satisfaction with the intervention could be evaluated in 10 patients (33%). The other 20 patients passed away or could not be reached for follow-up. Eight patients (80%) were satisfied or very satisfied and would recommend this intervention to other patients. Two patients were neutral	Hospital admission within 3 months Satisfaction of patients
Cheng et al. (40)	CHF patients (*n =* 82) Medical center	RCT	Comprehensive ACP intervention, 3 weeks	After the ACP intervention, no significant differences in posttest total, antibiotics, CPR, surgery, or ANH scores were observed between the groups (*p* > 0.05). However, in the experimental group, significant differences were observed between pretest and posttest total (z = -5.424, *p < * 0.001), antibiotics (z = -5.186, *p < * 0.001), CPR (z = -5.129, *p < * 0.001), surgery (z = -4.680, *p < * 0.001), and ANH (z = -4.952, *p < * 0.001) scores	Preferences for EOL care

CHF, chronic heart failure; DS-ACP, disease-specific advance care planning; ADAS, Advance Directive Attitude Survey; ACP, advance care planning; AD, advance directive; POLST, physician orders for life-sustaining treatment forms; SPIRIT-HF, sharing patients' illness representations to increase trust for heart failure; CPR, cardiopulmonary resuscitation; ANH, artificial nutrition and hydration; GOC, goals of care; DCS, the decisional conflict scale; DMC, the surrogate decision-making confidence scale.

**Table 3:** Summary of characteristics of qualitative results of the included studies.

**References**	**Participants and setting**	**Methodology/ methods**	**Phenomenon of interest**	**Theme**
Habal et al. (41)	CHF patients (*n =* 41) Medical center	Mixed methods Face-to-face interviews	The purposes of this study were to (1) determine patients' awareness, comprehension, and utilization of ACDs and (2) determine their knowledge of the process of cardiopulmonary resuscitation and their current resuscitation preference	Three themes: (1) awareness of ACDs; (2) knowledge of resuscitation options; (3) current resuscitation preference
Werdecker et al. (42)	CHF patients (*n =* 30) Medical center	Qualitative Grounded theory	The aim of the study was to analyze the perception of ACP consultations by patients with advanced heart failure	Four themes: (1) willingness to discuss ACP; (2) their illness; (3) death and dying, and the experienced; and (4) preferred role in healthcare decision-making

ACD, advance care directive; CHF, chronic heart failure; ACP, advance care planning.

### 2.4 Quality appraisal

The two independent reviewers applied the JBI Critical Appraisal Tool to evaluate eligible studies (31), which included assessment checklists for randomized controlled trials (RCTs), quasi-experimental studies (non-randomized experimental studies), and qualitative studies. Assessment checklists provided answers to all questions in the form of “Yes,” “No,” or “Unclear.” An all-“Yes” response represented high quality of the study; one or two “Unclear” or “No” responses represented medium quality; and more than two “Unclear” or “No” responses represented low quality. All the included studies were categorized into low, moderate, and high quality using this method. Any disagreements that arose during the data extraction and quality assessment process were discussed in depth until a consensus was reached.

### 2.5 Data synthesis and integration

As this review aimed to explore the different dimensions of a phenomenon of interest, a convergent segmentation method was used to synthesize and integrate quantitative and qualitative data (27, 31). Where available, statistical meta-analyses of the quantitative data were conducted, and Review Manager V.5.4 of Cochrane Collaboration was used to obtain combined effect estimates. In addition, *I*^2^ statistic and χ^2^ test were used to assess heterogeneity. When statistical aggregation was not feasible, a narrative approach was followed to summarize the results. For the results of the qualitative study, the JBI methodology was adopted and meta-aggregation methods were used for integration (31). The findings obtained from the included studies (level 1) were compiled into coherent statements. Subsequently, similarities in meaning among these findings were collected to establish categorization (level 2). Finally, by synthesizing these categories (level 3), a comprehensive collection of the synthesized findings was produced for use in evidence-based practice (31).

## 3 Results

### 3.1 Study selection

**Figure 1:**
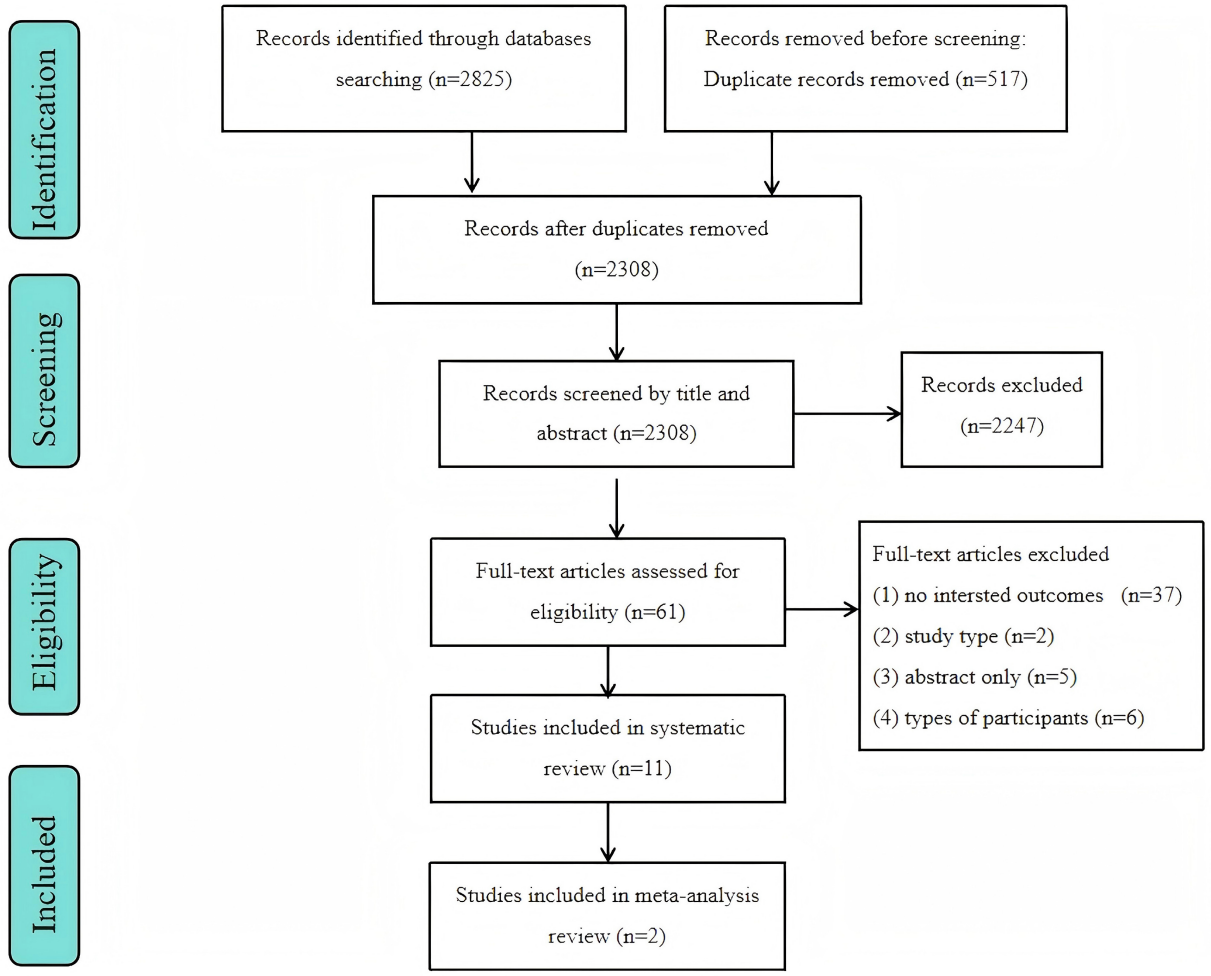
Preferred Reporting Items for Systematic Reviews and Meta-Analyses flow diagram of the study selection process.

A total of 2,825 articles were retrieved in our search. After removing 517 duplicates, the title and abstract of the remaining 2,308 papers were examined, which resulted in the selection of 61 studies. Then, the full text of these 61 studies was checked. Finally, 11 articles were included (32–42) (Figure 1).

### 3.2 Methodological quality

Among the quantitative studies included (*n* = 9), six studies were rated as medium quality, one as high quality, and two as low quality. There were five RCTs, two of which had missing information regarding the blinding of participants (32, 40) and four lacked information on the blinding of treatment assignment (32, 35, 37, 40). The remaining three studies were classified as quasi-experimental studies and one as mixed study. Four studies lacked a control group (33, 34, 38, 39). Two studies did not provide information on whether participants received comparable treatment or care in addition to the specific exposure or intervention being investigated (38, 39). One study lacked information regarding multiple outcome measurements (39). All included qualitative studies (*n* = 2) were rated as moderate quality because the authors did not clearly articulate their theoretical frameworks, cultural orientations, and the potential impact of these factors (41, 42).

### 3.3 Characteristics of the included studies

The core characteristics of the included studies were summarized and presented. A total of 11 articles, whose publication year spanned from 2011 to 2024, were included in this review. Study designs encompassed five two-arm RCTs (*n* = 5) (32, 35–37, 40), one mixed-method design combining a one-arm pretest–posttest approach with a qualitative component (38), two one-arm pretest–posttest design (33, 34), one one-arm posttest design (39), and two qualitative studies (41, 42). A total of 2,809 participants were recruited in these studies. The sample size varied between 20 and 1,894 participants, all of whom were sourced from medical centers. Study samples had an average age of 53–83 years, and all the samples were from hospital settings.

### 3.4 Synthesis of quantitative evidence

A meta-analysis can be conducted to examine the impact of interventions on EOL care preferences as it allows for the analysis of consistent data across studies (32, 35). Other outcomes, including ACP completion, satisfaction, readiness, attitude, knowledge, ACP engagement, ACP self-efficacy, dynamics for EOL preferences, congruence, and EOL care preferences. These results could not be statistically summarized and were therefore represented through narrative integration.

#### 3.4.1 ACP-related completion

Three studies showed ACP-related completion after ACP intervention (32–34). In one study, a significant proportion of participants (94.3%) who finished the disease-specific advance care planning (DS-ACP) had a health directive in place, whereas only 24.8% of those who did not complete the DS-ACP process had a health directive (32). Another study demonstrated that after the intervention, 62% of patients had completed either an AD or physician orders for life-sustaining treatment (POLST) form within a 6-month period, as opposed to 51% who did that before the intervention. Furthermore, there was an increase in the number of patients completing a POLST form within 6 months of the intervention, from 13 patients (35%) to 19 patients (51%) (34). One showed an increase in the number of participants completing ADs (28% vs. 47%) (33).

#### 3.4.2 Satisfaction

Only one study reported satisfaction regarding ACP interventions (39). The results indicated a high level of satisfaction by patients, with 80% of patients expressing either satisfaction or very high satisfaction and stating that they would recommend the intervention to other patients.

#### 3.4.3 Readiness

A statistically significant increase was observed in patient readiness to engage in ACP from before the intervention to after the intervention, with a change score of +0.53 (*p* < 0.01) (38). However, at the 1-month follow-up, it decreased almost back to the level before the panel visit (change score of 0.52; *p* < 0.01) (38).

#### 3.4.4 Attitude

One study used a revised version of the Advance Directive Attitude Survey (ADAS) to evaluate patients' attitudes toward ACP interventions. The findings revealed a notable increase in ADAS mean scores, from 57% at baseline to 80% after 3 months (33). A statistically significant enhancement was observed in patients' readiness to engage in ACP from before the intervention to after the intervention, with a change score of +0.53 (*p* < 0.01) (36).

#### 3.4.5 Knowledge

One study used ACP Knowledge Questionnaire to evaluate ACP knowledge and found that participants in the intervention arm had higher mean knowledge scores compared with control participants (4.1 ± 1.4 vs. 3.0 ± 1.5; *p* < 0.001) (35).

#### 3.4.6 ACP engagement

Only one study addressed ACP engagement, not through specific data points but rather by noting that participants in the intervention group were more inclined to report having conversations about goals of care (GOC) with their healthcare providers (35).

#### 3.4.7 Dynamics for end-of-life preferences

One study used a self-efficacy questionnaire to assess patients' self-efficacy levels and found that those with higher self-efficacy were more likely to choose aggressive EOL care (37). Conversely, it reported a progressive increase in the percentage of patients who changed their EOL care preferences compared with the baseline figures (37). In addition, the same study found that EOL preferences are influenced by other factors, suggesting that older patients have a lower level of interest in preferences for aggressive EOL care as their preferences are more influenced by factors such as financial constraints, proper knowledge of prognosis, and conflicts in decision-making (37).

#### 3.4.8 AD-related outcomes

A study that used the self-efficacy questionnaire as an assessment tool showed that there was no statistically significant overall change in patient self-efficacy (with a change score of +0.29; *p* = 0.11). Notably, 1 month after the group intervention, a decline in self-efficacy was observed, with levels falling below those recorded before the group intervention (change score −0.40; *p* < 0.05) (38).

#### 3.4.9 Congruence

Two studies examined the consistency in preferences by patients and their families/doctors. One study revealed that the intervention group showed a higher degree of alignment with respect to GOC. Specifically, individuals in the intervention group were approximately nine times more likely to achieve congruence in GOC compared with those in the control group. However, the difference observed between the two groups did not reach statistical significance (*p* = 0.064) (36). Another study reported that the agreement between clinicians' and patients' ACP preferences was higher in the intervention group than in the control group (35).

#### 3.4.10 Preference of EOL care

**Figure 2:**
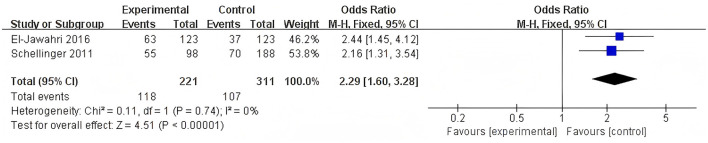
Forest plot of pooled results for preference of EOL care.

A combined analysis of two studies showed a significant impact of the intervention on the selection of EOL care preferences (Figure 2). Specifically, the mean difference in EOL care preference was 2.29, with a 95% confidence interval (CI) ranging from 1.60 to 3.28. However, it should be noted that the *p*-value was 0.75, indicating no statistical significance, and the *I*^2^ statistic was 0%, suggesting no heterogeneity between the two studies (32, 35).

### 3.5 Synthesis of qualitative evidence

Using a meta-aggregation process of the qualitative evidence, three key themes were identified: insufficient information, positive impact, and clashing perspectives. ACP-related knowledge was categorized under the insufficient information theme. ACP engagement strategies and gain benefits were two subthemes identified in the positive impact theme. Role of family and decision-making are two subthemes identified within the clashing perspectives theme (Table 4).

**Table 4:** Meta-aggregation of qualitative findings.

**Findings (level 1)**	**Categories (level 2)**	**Synthesized findings (level 3)**
Most participants did not know ACDs (41) (U) Gives me a clear picture of what I want and what I do not want (42) (U) Few participants reported having discussed the ACP-related knowledge with their physician (41) (U) Few participants were unable to clearly describe the purpose and/or process of resuscitation (41) (U) Few participants did not know what cardiopulmonary resuscitation was (41) (U) Few participants had documented their resuscitation preference (41) (U) Considerable skepticism about the usefulness of advance planning as they had not been involved in previous healthcare (42) (U)	ACP-related knowledge	Insufficient information
The higher the level of participation in preventive measures, diseases, and deaths, the higher the willingness to engage in prior consultation planning (42) (U) The better you understand your condition, the more you will be willing to plan ahead (42) (U) The success of an open-ended ACP consultation is determined by nothing more than the atmosphere, the time allotted, the respectful way in which mutual communication takes place, trust, and the orientation to the patient's individual needs (e.g., information needs, concerns, and fears) (42) (U) ACP can be expressed as an open-ended process and must have the will of the individual (42) (U) Most participants favored discussing them (41) (U) Many of these participants would have preferred to discuss them early on after the diagnosis of HF, before complications arise (41) (U)	ACP engagement strategies	Positive impact
Participants feel comfortable at home. It is better than being in the hospital (42) (U) Someone to listen to me so I can let my ideas run wild (42) (U) He explained it all very well to me and I was relieved that I was told it was all so clear (42) (U)	Gain benefits	
Families will question patients' decisions that are surprising to them or seem unreasonable to them (42) (U) Relatives are involved in the decision-making process by the patient as a peer in order to obtain expert information or clarification of substantive issues from a professional counselor (42) (U) Involvement of relatives leads to conflict in some patients due to different goals (42) (U)	Role of family	Clashing perspectives
The greater the desire for shared decision-making and the experience of participating in the healthcare system, the clearer the ideas about prevention and the desire for self-determination for future decisions (42) (U) Using the framework of ACP counseling and communication with relatives to make specific decisions (42) (U) Nearly half of the participants stated they would want their negative treatment when their condition worsens (41) (U) Creating spaces for open communication with relatives also contributes to the decision-making process (42) (U) Most participants were able to identify someone whom they would choose as their SDM (41) (U)	Decision-making	

U and C represent the levels of credibility for the findings: U represents “unequivocal” evidence, C represents “credible” evidence, and N represents “not supported” evidence.

SDM, substitute decision maker; ACDs, advanced care directives; ACP, advanced care plan.

#### 3.5.1 ACP-related knowledge

The majority of participants did not know ACP and were even rather skeptical about the usefulness of ACP (41, 42). Some participants thought that an ACP intervention gives them a clear idea of what they want and do not want (42).

#### 3.5.2 ACP engagement strategies

The majority of participants expressed a willingness to engage in ACP discussions and believed that they obtained higher levels of knowledge about the disease after the discussion than earlier and showed a strong willingness of patients to participate in the ACP (41, 42). In addition, the success of an ACP discussion was found to be influenced by various factors, such as the atmosphere, time allotted, way of communication, trust, and orientation to the patient's individual needs (42).

#### 3.5.3 Gain benefits

A number of participants reported that the medical staff explained the ACP clearly and made them feel comfortable and at ease (42). Some participants expressed that medical staff need to listen to them so that they can express their wild ideas (42).

#### 3.5.4 Role of family

Besides participants, their families were also involved in ACP decision-making to obtain expert information or clarification of significant issues from professional advisers and to make unanimous decisions (42). However, when a participant makes a decision that surprises their family or seems unreasonable to them, the family questions the decision and potentially creates conflict (42).

#### 3.5.5 Decision-making

Participants felt that creating spaces for open communication with relatives regarding ACP counseling helped their decision-making process (41, 42). The stronger the desire for co-decision-making, the stronger the desire for self-determination over future decision-making. After ACP intervention, participants expressed that they were able to identify someone whom they would choose as their substitute decision maker (41).

### 3.6 Integration of quantitative and qualitative evidence

The combined results of the quantitative and qualitative evidence were generally consistent, and the results of the three-part summary are presented in detail in Table 5. The positive perceptions of participants toward the ACP intervention, as reflected in the qualitative evidence, provided insights into the significant enhancements observed in various ACP outcomes reported in the quantitative evidence.

**Table 5:** Integration of quantitative evidence and qualitative evidence.

**Quantitative results**	**Qualitative finding (categories)**	**Aggregation**
ACP completion In intervention group participants, 94.3% had a health directive (32) The number of participants who completed ADs/POLST increased (33, 34)	Gain benefits ACP-related knowledge	Empowerment
Satisfaction The majority of patients (80%) expressed satisfaction and would recommend this intervention to other patients (39)		
Readiness Eight out of the 10 items comprising the readiness scale significantly increased from pre- to-post-group visit levels (38) Patient readiness for ACP improved significantly post-group visit but declined to pre-group levels by the 1-month follow-up (38)		
Attitude Average scores on the ADAS increased from 57% to 80% (33) Almost all participants in the intervention group reported a positive experience (36)		
Knowledge Intervention group participants had higher mean knowledge scores (4.1 ± 1.4) than controls (3.0 ± 1.5) (35)		
ACP engagement Intervention group participants were more likely to report GOC conversations with the healthcare provider (35)		
ACP self-efficacy Patient self-efficacy remained largely unchanged and decreased below pre-group levels at the 1-month assessment (38)		
Dynamics for EOL preferences Patients with higher self-efficacy were more likely to prefer aggressive EOL care (37) The proportion of patients changing their EOL care preferences relative to the baseline gradually increased (37) Patient preference for aggressive EOL care was lower for older patients (37) Preferences were heavily influenced by finances, prognostic understanding, and decisional conflict (37)	ACP engagement strategies	Obstacles and facilitators
Congruence The intervention group showed higher improvement in dyad congruence on GOC (36) Intervention group participants were nearly nine times as likely as controls to achieve congruence in patients' GOC (36) The concordance of clinicians' and patients' ACP preferences was higher in the intervention group than in the control group (35)	Role of family Decision-making	Decision-centered
Preference of EOL care 56.1% of ACP completers were enrolled in hospice, compared with 37.2% of those who did not complete DS-ACP (32) Most patients (78%) did not want to be readmitted and preferred to die at home (39) Most patients were readmitted during follow-up, but did they undergo invasive diagnostic procedures (39) Significant differences were observed in the experimental group between pre- and posttest scores for antibiotics, CPR, surgery, and ANH (40) A higher number of participants in the intervention arm preferred comfort care compared with those in the control arm (35) After the intervention, a higher number of participants in the intervention group preferred to decline CPR and intubation than those in the control group (35) 64% patients changed their preference of EOL care at least once through the study period (40)		

ACP, advance care planning; DS-ACP, disease-specific advance care planning; AD, advance directive; POLST, physician orders for life-sustaining treatment forms; ADAS, Advance Directive Attitude Survey; GOC, goals of care; EOL, end of life; CPR, cardiopulmonary resuscitation.

#### 3.6.1 Empowerment

Quantitative and qualitative studies have consistently stated that ACP interventions are beneficial in promoting patients' understanding of ACP-related knowledge. In this study, quantitative data showed that ACP interventions significantly increase participants' completion (32–34) while also improving their engagement in ACP (35). In addition, quantitative data also suggested that the ACP intervention increases patient satisfaction and a sense of good experience during the intervention process, which depends largely on the professionalism of the ACP intervention. Besides, qualitative data suggested that ACP enables people to have a clear understanding of what they want and to be comfortable discussing it with medical staff/families to achieve the most satisfactory outcome (41, 42).

#### 3.6.2 Obstacles and facilitators

Both quantitative and qualitative studies highlighted facilitators and obstacles to ACP preferences (37, 41, 42). Quantitative data showed that patients with higher self-efficacy are more likely to prefer aggressive EOL care and that age, economic status, knowledge of the disease, and decision-making conflicts are likely to influence patients' EOL preferences (37). However, participants' EOL preferences are not static; rather, they are more likely to change over time (37). Quantitative data also showed other subjective factors such as environment, timing, and way of communication style, but the most fundamental factor was the individual's willingness (42).

#### 3.6.3 Decision-centered

Quantitative and qualitative studies emphasized decision-centered preference selection among clinicians, participants, and families. Quantitative data showed that after participants received ACP intervention, decision-making consistency between participants and both physicians and family members was found to be significantly higher than in the control group, and the majority of participants were more inclined to receive comfort care after the ACP intervention, reducing the invasive quality of care and enhancing patient quality of life (35, 36, 39, 40). Qualitative and quantitative evidence complemented each other, with quantitative data directly reflecting outcomes in this integration and qualitative data reflecting the complexity of the decision-making process, such as the conflict and mutual questioning of decision-making between patients and families, and the difficulty for patients in choosing an agent (42).

## 4 Discussion

### 4.1 Major findings

In this study, JBI's MMSR framework was used to synthesize both quantitative and qualitative evidence. This approach facilitated to achieve a comprehensive understanding of the effectiveness of ACP and the experiences of the participants (27). There were differences in the quality of quantitative studies as all prospective studies did not have a control group. The quality of qualitative studies was rated as moderate as these two studies lacked a theoretical framework and also due to the researcher's reflective and self-critical nature of the study.

The overall results showed that the integration of quantitative and qualitative research is both supplementary and consistent, with quantitative research as an etic perspective providing an objective outcome and qualitative research as an emic perspective providing process understanding. This helps demonstrate that the ACP intervention is a practical and effective approach to promoting ACP empowerment (including knowledge and attitudes) and decision-making for participants. Quantitative results in this study showed a significant increase in the number of ACP/AD completions and ACP knowledge scores in the intervention group (32, 35). This finding suggests that ACP intervention is effective in increasing patient understanding and engagement with the pre-established healthcare plan. This is consistent with the findings of the existing research. For example, Liu et al. (43) have found that a gamified ACP intervention significantly improves patients' ACP knowledge and completion rates. Qualitative results in this study add process to this finding and show that the medical staff provided clear explanations about ACP during the intervention, thus enhancing the patients' understanding of the ACP (42). Previous studies (44) have agreed that community populations show a low level of ACP participation. Therefore, based on the results of this review, promotion and explanation of ACP should be carried out at all levels of healthcare as the professional guidance of medical staff can have a positive effect on different populations. Participants in a qualitative study emphasized the importance of a respectful atmosphere and open communication (42), which may be one of the reasons for the high satisfaction rate reported in quantitative results (39). High-quality conversations are not possible without a good communication climate, and several cross-sectional studies (45, 46) now point to cursory, brief, and unprofessional communication as a significant impediment to achieving high patient satisfaction. Therefore, the quality of ACP interventions by medical staff relies not only on the content of communication but also on communication style and patient experience. Medical staff should be trained in communication skills to facilitate participation in ACP discussions. Quantitative results indicated a high rate of family involvement in decision-making and a significant increase in goal agreements between patients and families after ACP intervention (35, 36). However, one qualitative study also revealed the potential for conflict resulting from family involvement (42). Families may prefer aggressive treatment, whereas patients prefer comfort care. This inconsistency in decision-making may stem from emotional factors, differences in information about the condition, and cultural and social pressures. Therefore, healthcare professionals should train family members in communication skills and hospice knowledge to help them better understand the patient's wishes and values, to reduce emotional and practical pressures, to make clear that the role of the family is that of a supporter rather than a decision-maker, and to ensure that the patient's decision-making is autonomous and that the patient has the right to die with dignity, with the least amount of pain and suffering possible (47). In this study, we found that patients' ACP self-efficacy and readiness increased significantly after the intervention, but decreased to pre-intervention levels after 1 month (38). In contrast, previous studies have reported a significant increase in patients' self-efficacy and readiness after ACP intervention, but without returning to pre-intervention levels after a specific time period (43, 48, 49). Qualitative studies have further revealed patients' skepticism about the long-term usefulness of ACP (42). Therefore, it is possible that the long-term effectiveness of ACP intervention was affected by the skepticism and lack of knowledge of some of the participants.

Qualitative research and quantitative research complement each other, with the former revealing the mechanisms of action and contextual factors of interventions through in-depth analyses of participants' experiences and perspectives and the latter assessing the effectiveness of interventions through statistical analyses. However, in this study, qualitative research was not directly tested using quantitative research in some aspects, which provides a potential direction for future research. Regarding the timing of ACP discussions, qualitative findings suggested that many participants like to discuss ACP during the early stage of the disease (41), whereas quantitative studies did not explicitly test this. This finding is highly consistent with clinical practice recommendations that discussing ACP during the early stage of the disease significantly improves patient engagement and quality of decision-making. Ideal implementation of ACP should be an ongoing and dynamically adapted process that emphasizes a prospective and systematic approach rather than an acute and *ad hoc* one. For patients with CHF, the best practice is to initiate ACP at an early, stable stage of the disease, rather than waiting until the disease worsens. This early intervention helps build a therapeutic relationship based on care, trust, and long-term cooperation and creates favorable conditions for adequate communication and continuous adjustment of the patient's treatment preferences (50). In addition, qualitative findings revealed important insights into patients' preferences for EOL care, such as the desire to avoid hospital readmission and dying at home, and the dynamic nature of preferences (35). However, quantitative results, including a non-significant *p*-value for EOL care preferences (*p* = 0.75) and wide confidence intervals (1.60–3.28), suggest a significant uncertainty about the effect of the intervention, and these results should be interpreted with caution. Future studies should employ larger sample sizes and standardized intervention protocols to clarify the effect of interventions on hospice preferences.

Meta-analysis revealed that the ACP intervention for older adult individuals with CHF significantly enhances participants' satisfaction with ACP, readiness for ACP, knowledge related to ACP, engagement in ACP behaviors, and ultimately a higher completion rate of ACP. Meta-aggregation showed that participants generally lack ACP-related knowledge but have a positive attitude, and participants believe that ACP intervention provides a suitable opportunity to facilitate shared decision-making in the family and explain the relevant variables affecting ACP. These observations were consistent with the primary sources of the theory of planned behavior where attitude (i.e., evaluation of behavior and affective tendencies), subject norms (i.e., expectations and perceptions of others about particular behaviors), and perceived behavioral control (i.e., individuals' perceptions of their ability to successfully perform particular behaviors) shape an individual' s behavioral intentions (i.e., intention or desire to perform an act) and behavioral outcomes (i.e., behavior that is actually exhibited by an individual in a given situation) (51). The results of the present study are consistent with this theoretical model, which emphasizes that ACP participation is a dynamic process rather than a static event that fluctuates in response to changes in internal and external factors, such as self-efficacy maintenance and complexity of family involvement. These findings provide an important basis for optimizing ACP interventions and highlight the directions for future research. Future research should focus on the long-term effects of ACP interventions. Furthermore, intervention strategies targeting family engagement need to be developed to reduce conflict and increase consistency in decision-making.

### 4.2 Strengths and limitations

The advantage of this study lies in the comprehensive and thorough analysis of quantitative and qualitative data using the MMRS to elucidate the impact of ACP intervention on CHF participants and their experiences. Because of the limited number of studies on this subject, all identified studies were included in the literature, including one qualitative study in German. A consensus was established on the integration of quantitative and qualitative evidence, which subsequently enhanced the credibility of the findings regarding the effectiveness of ACP and engagement levels associated with it.

However, this review has some limitations. First, an in-depth search of the available literature was conducted, but relevant studies might have been overlooked due to the inclusion criterion limiting the study language to English and German. Second, the strength of the evidence was weakened as all prospective studies used a single-group design. In addition, the limited number of qualitative studies and the absence of a theoretical framework guiding the included qualitative evidence further compromised the level of evidence. Third, participants in the included studies were recruited exclusively from hospital settings. Thus, it is uncertain whether the findings of this study are applicable to older adult patients with CHF at home or in the community. Furthermore, the primary limitations of conducting a meta-analysis were the small number of included studies and the inability to formally assess publication bias (e.g., funnel plots or Egger's test) due to insufficient statistical power. This may affect the robustness and generalizability of our findings. Therefore, future studies should include a higher number of studies to address this limitation.

## 5 Conclusion

Comprehensive evidence from studies with quantitative and qualitative data suggests that ACP intervention is an effective method to make participants aware of healthcare values and goals, thereby improving their outcomes. Since older patients with CHF lack ACP-related knowledge and an opportunity to start ACP discussions with clinicians or families, they miss a valuable chance to have a serious and complete discussion about ACP. Therefore, broader and more rigorous studies, especially prospective studies, are required to analyze the effects of ACP on older patients with CHF using rigorous study designs and in different cultures. In addition, future studies need to extend the follow-up period to explore the possibility of and reasons for changes in the variables of ACP-related outcomes over time.

## Data Availability

The original contributions presented in the study are included in the article/Supplementary material, further inquiries can be directed to the corresponding authors.
