# Supplementary material for: Implementation and effectiveness of advance care planning in hospitalized older adults with chronic heart failure: a mixed-methods systematic review and meta-analysis
Source: Front Med (Lausanne). 2025 Apr 29;12:1566977. doi: 10.3389/fmed.2025.1566977 (PMC12069042; doi:10.3389/fmed.2025.1566977)
Supplement: Supplementary file 1 [file Data_Sheet_1.pdf]

Appendix 1
Search date: 29/10/2024

Pubmed
(((Advance Care Planning[MeSH Terms]) OR (Advance Health Care Planning[Title/Abstract] OR Advance Medical Planning[Title/Abstract] OR Medical Planning, Advance[Title/Abstract] OR Planning, Advance Medical[Title/Abstract] OR Advance Directives[Title/Abstract] OR Living Wills[Title/Abstract] OR Terminal Care[Title/Abstract])) AND (("Heart Failure"[Mesh]) OR (Cardiac Failure[Title/Abstract] OR Heart Decompensation[Title/Abstract] OR Decompensation, Heart[Title/Abstract] OR Congestive Heart Failure[Title/Abstract] OR Heart Failure, Congestive[Title/Abstract] OR Heart Failure, Right-Sided[Title/Abstract] OR Heart Failure, Right Sided[Title/Abstract] OR Right-Sided Heart Failure[Title/Abstract] OR Right Sided Heart Failure[Title/Abstract] OR Heart Failure, Left-Sided[Title/Abstract] OR Heart Failure, Left Sided[Title/Abstract] OR Left-Sided Heart Failure[Title/Abstract] OR Left Sided Heart Failure[Title/Abstract] OR Myocardial Failure))) AND (("Aged"[Mesh]) OR (Elderly[Title/Abstract]))

CINAHL and Medline
S1 "Advance* Care plan*"
S2 "Anticipatory care plan*"
S3 "Future care planning"
S4 (MH "Advance Care Planning")
S5 "Advance Care Planning"
S6 "Living Wills"
S7 (MH "Advance Directives+") OR (MH "Living Wills")
S8 "Advance Directives"
S9 "Resuscitation Orders"
S10 "Medical treatment order"
S11 "Statement of wishes"
S12 "Medical directive"
S13 "End of life discuss*"
S14 "End of life conversation*"
S15 "End of life decision*"
S16 "End of life plan*"
S17 "End of life preference*"
S18 "Advance* Medical plan*"
S19 "Advance* Statement*"
S20 S1 OR S2 OR S3 OR S4 OR S5 OR S6 OR S7 OR S8 OR S9 OR S10 OR S11 OR S12 OR S13 OR S14 OR S15 OR S16 OR S17 OR S18 OR S19
S21 (MH "Aged+") OR(Aged or Aging OR Old* OR Elderly OR Senior OR Elder)
S22 (MH "Heart failure")
S28 S20 AND S21 AND S22

Web of science

((TS=("Advance* care plan*" OR "Anticipatory care plan*" OR "Future care planning" OR (MH "Advance Care Planning") OR "Advance Care Planning" OR "Living Wills" OR (MH "Advance Directives+") OR (MH "Living Wills") OR "Advance Directives" OR "Resuscitation Orders" OR "Medical treatment order" OR "Statement of wishes" OR "Medical directive" OR "End of life discuss*" OR "End of life conversation*" OR "End of life decision*" OR "End of life plan*" OR "End of life preference*" OR "Advance* Medical plan*" OR "Advance* Statement*")) AND TS=(Aged or Aging OR old* OR Elderly OR Senior OR Elder)) AND TS=(Heart failure)

CNKI

Heart failure * Older * Advance care planning

Embase

#4 #1 AND #2 AND #3

#3 'Heart failure'/exp

#2 'Aged' OR 'Aging' OR 'Old*' OR 'Elderly' OR 'Senior' OR 'Elder'

#1 'Advance care planning'/exp OR '(Advance* care adj plan*)' OR 'Statement of wishes' OR 'Terminal care' OR 'Living wills' OR 'Future care planning' OR 'Advance directives' OR 'Resuscitation orders' OR 'Medical treatment order' OR 'Medical directive' OR 'End of life discuss*' OR 'End of life conversation*' OR 'End of life decision*' OR 'End of life plan*' OR 'End of life preference*' OR 'Advance* Medical plan*' OR 'Advance* Statement*'

Cochrane

#1 (Advance care planning):ti,ab,kw OR (Advance Directive):ti,ab,kw OR (Living will):ti,ab,kw OR (advance* NEAR/3 plan*):ti,ab,kw OR (Future care planning):ti,ab,kw

#2 (Anticipatory care plan*):ti,ab,kw OR (End of life NEXT (Fiscuss* or conversation* or Decision* or Plan* or Preference*)):ti,ab,kw OR (Medical treatment order):ti,ab,kw OR (Statement of wishes):ti,ab,kw OR (Medical directive):ti,ab,kw

#3 (Advance* NEXT (medical plan* or statement*)):ti,ab,kw

#4 Advance care planning

#5 Advance Directive

#6 Living will

#7 (Heart Faliure):ti,ab,kw

#8 (Aged):ti,ab,kw OR (Elderly):ti,ab,kw

#9 #1 or #2 or #3 or #4 or #5 or #6

#10 #7 AND #8 AND #9

Appendix 2

Table 2a. Methodological quality of included randomized controlled trial

	Schellinger et al, 2011	El-Jawahri et al, 2016	Metzger et al, 2016	Malhotra et al, 2020	Cheng et al, 2024
1. Was true randomization used for assignment of participants to treatment groups?	Unclear	Yes	Yes	Yes	Yes
2. Was allocation to treatment groups concealed?	Unclear	Yes	Yes	Yes	Yes
3. Were treatment groups similar at the baseline?	Yes	Yes	Yes	Yes	Yes
4. Were participants blind to treatment assignment?	No	Yes	Yes	Yes	No
5. Were those delivering treatment blind to treatment assignment?	No	No	Yes	No	No
6. Were outcomes assessors blind to treatment assignment?	Unclear	Unclear	Yes	Yes	Yes
7. Were treatment groups treated identically other than the intervention of interest?	Yes	Yes	Yes	Yes	Yes
8. Was follow up complete and if not, were differences between groups in terms of their follow up adequately described and analyzed?	Yes	Yes	Yes	Yes	Yes
9. Were participants analyzed in the groups to which they were randomized?	No	Yes	Yes	Yes	Yes
10. Were outcomes measured in the same way for treatment groups?	Yes	Yes	Yes	Yes	Yes
11. Were outcomes measured in a reliable way?	Yes	Yes	Yes	Yes	Yes
12. Was appropriate statistical analysis used?	Yes	Yes	Yes	Yes	Yes
13. Was the trial design appropriate, and any deviations from the standard RCT design (individual randomization, parallel groups) accounted for in the conduct and analysis of the trial?	No	Yes	Yes	Yes	Yes
Overall quality	Low	Moderate	High	Moderate	Moderate

Appendix 2

Table 2b. Critical appraisal results for included quasi-experimental studies or mixed methods studies or no randomized controlled trial

	Evangelista et al, 2012	Sadeghi et al, 2016	Ahluwalia et al, 2021	Coster et al, 2022
1. Is it clear in the study what is the 'cause' and what is the 'effect' (i.e. there is no confusion about which variable comes first)?	Yes	Yes	Yes	Yes
2. Were the participants included in any comparisons similar?	Yes	Yes	Yes	Yes
3. Were the participants included in any comparisons receiving similar treatment/care, other than the exposure or intervention of interest?	Yes	Yes	Unclear	Unclear
4. Was there a control group?	No	No	No	No
5.Were there multiple measurements of the outcome both pre and post the intervention/exposure?	Yes	Yes	Yes	No
6. Was follow up complete and if not, were differences between groups in terms of their follow up adequately described and analyzed?	Yes	Yes	Yes	Unclear
7. Were the outcomes of participants included in any comparisons measured in the same way?	Yes	Yes	Yes	Unclear
8. Were outcomes measured in a reliable way?	Yes	Yes	Yes	Yes
9. Was appropriate statistical analysis used?	Yes	Yes	Yes	Yes
Overall quality	Moderate	Moderate	Moderate	Low

Appendix 2

Table 2c. Critical appraisal results for included qualitative studies

	Habal et al (2011)	Werdecker et al(2019)
1. Is there congruity between the stated philosophical perspective and the research methodology?	Yes	Yes
2. Is there congruity between the research methodology and the research question or objectives?	Yes	Yes
3. Is there congruity between the research methodology and the methods used to collect data?	Yes	Yes
4. Is there congruity between the research methodology and the representation and analysis of data?	Yes	Yes
5. Is there congruity between the research methodology and the interpretation of results?	Yes	Yes
6. Is there a statement locating the researcher culturally or theoretically?	No	No
7. Is the influence of the researcher on the research, and vice- versa, addressed?	Yes	Yes
8. Are participants, and their voices, adequately represented?	Yes	Yes
9. Is the research ethical according to current criteria or, for recent studies, and is there evidence of ethical approval by an appropriate body?	Yes	Yes
10. Do the conclusions drawn in the research report flow from the analysis, or interpretation, of the data?	Yes	Yes
Overall quality	Moderate	Moderate
